# Performance of an emergency department observation unit protocol in reducing length of stay for acetaminophen overdose: a retrospective study

**DOI:** 10.1186/s12245-018-0210-y

**Published:** 2018-11-16

**Authors:** Dilin Tang, Wui Ling Chan, Dong Haur Phua

**Affiliations:** Emergency Department, Tan Tock Seng Hospital, Emergency Department Level B1 11 Jalan Tan Tock Seng, Singapore, 308433 Singapore

**Keywords:** Emergency medicine observation ward, Acetaminophen overdose, Length of stay

## Abstract

**Introduction:**

Acetaminophen is one of the most common causes of poisoning among developed countries. The emergency department observation unit (EDOU) has been increasingly used in the management of various conditions to reduce hospitalisation but its efficacy in not well studied in management of poisoned patients. In this study, we aim to study the effectiveness of our EDOU in the management of acetaminophen overdosed patients.

**Results:**

Medical records of patients admitted from the emergency department from 2012 to 2016 for acetaminophen overdose were reviewed. One hundred ninety-five patients presenting with acetaminophen overdose were admitted to the EDOU while 184 were admitted to the general ward. Of these, 27 patients admitted to EDOU did not meet the admission criteria for it while 71 patients who met EDOU criteria were admitted to the ward instead. For patients who fulfilled EDOU admission criteria, median length of stay for EDOU patients was 23 h (IQR 19–24) while that for those admitted to the ward was 66 h (IQR 62.5–88.3).

**Conclusion:**

The EDOU is a safe alternative to hospitalisation for patients presenting with acetaminophen poisoning. It is also associated with a shorter length of stay for such patients. Further studies need to be done to assess the cost-effectiveness of EDOU for management of patients presenting with acetaminophen overdose.

## Introduction

Acetaminophen is one of the most common causes of poisoning in developed countries [1, 2]. Similarly in Singapore, it is one of the most common drugs that is overdosed by both children and adults [3–5]. It is a common cause of liver failure in Europe and North America, which is associated with significant morbidity and mortality [6]. The mainstay of treatment is the timely administration of intravenous *N*-acetylcysteine (NAC) [7]. The standard treatment protocol for acetaminophen overdose is a 21-h course of intravenous (IV) NAC infusion. A previous study found that most general ward admissions secondary to drug overdose lasted 2 to 3 days only and were also largely managed for psychosocial rather than just medical issues [5].

The Emergency Department Observation Unit (EDOU) at our institution is a protocol-driven short-stay ward for patients with selected diagnoses to undergo further investigations and management for a defined time period up to 48 h. EDOUs are increasingly used to avoid hospital admissions in patients and may reduce the length of stay and cost of stay while providing high-quality healthcare [8–10]. Its role in management of poisoned patients is not well studied, but they may be used to extend the evaluation period for patients potentially needing inpatient admission; and also facilitate the administration of short-term interventions [11]. There is currently limited literature on the use of EDOU protocols for acetaminophen ingestion [12, 13].

In our institution, our emergency department (ED) developed a multi-disciplinary protocol for the treatment of acetaminophen in the EDOU. This protocol involves the emergency physicians (EP), toxicologists, nurses, psychiatrists, and medical social workers (MSWs). Together, they evaluate the patient for the appropriateness of 21-h IV NAC therapy and the progress under aforementioned therapy, as well as the results of further investigations, to determine the patient disposition to inpatient admission, further psychiatric workup or home discharge. The protocol provides different management strategies in terms of indications for IV NAC and evaluation of serum acetaminophen levels for single dose, staggered dose, sustained preparation, late presenter, and acetaminophen overdose of unknown timing.

In this study, we aim to describe our experience with our EDOU protocol over a 5-year period from 1 January 2012 till 31 December 2016 and assess its utilisation. We also aim to determine its effectiveness in terms of reducing hospital admissions and length of stay while providing the appropriate standard of care.

## Methods

This was a retrospective review of all patients admitted from the ED with a diagnosis of acute paracetamol poisoning from 1 January 2012 till 31 December 2016 to either the general ward or EDOU.

### Setting

The setting was that of a 1400-bed tertiary care hospital in Singapore. The EDOU is a 36-bed enclosed observation unit within the ED, staffed by two to three nurses and one doctor on each shift, under the oversight of an EP. Doctor rounds with the senior doctor are scheduled three times daily, to assess patients for further management or discharge. Serial investigations and treatment, including cardiac monitoring and laboratory testing, may be performed on the patients in the EDOU. The senior EP makes all decisions regarding admission to the EDOU and the patient’s final disposition.

The study was reviewed and approved by the Institutional Review Board (reference no: 2017/008700). Waver of patients’ consent was granted by the ethics board for this study due to minimal risk to patients.

### Acetaminophen overdose protocol

The protocol was implemented since 2010. All asymptomatic and hemodynamically stable patients were attended to and managed in the intermediate acuity care area of the ED by medical officer with oversight of a senior EP. A thorough history and physical examination was carried out for every patient. Important questions include the time of overdose, amount and type of drug, reason for overdose, as well as the presence of any other co-ingested substances or comorbidities were asked. Standard initial blood tests include a full blood count, renal panel, liver function test and coagulation studies and electrocardiogram were performed. For single ingestions, serum acetaminophen levels were taken at 4 h post-ingestion or as soon as possible thereafter and plotted on Rumack-Mathew nomogram that defines toxicity acetaminophen level as above 150 mg/dl at 4 h. For staggered ingestions, delayed presentations and unclear history time of ingestion, decision for antidote treatment is at the discretion of the supervising senior physician.

All supervising physicians have the option of admitting these patients to either the EDOU or a general ward under the medical discipline. The eligibility criteria for patients to be admitted to the EDOU acetaminophen overdose protocol is listed in Table 1.

**Table 1:** Inclusion and exclusion criteria of EDOU pathway for acetaminophen poisoning

Inclusion criteria	
Clinically significant history for overdose	
Single overdose	
Staggered overdose	
Overdose with unknown time	
Sustained release formulation	
Overdose with gastro-motility delaying agents	
Late presenter (more than 24 h post-ingestion)	
Exclusion criteria	
Unstable patients (symptoms, vital parameters and mental state)	
Children less than 16 years old	
Significant co-morbidities	
Significant co-ingestant	
Already has criteria to refer for transplant consideration	

Standard treatment in the protocol was a 21 h IV NAC infusion as well as management of any symptoms that the patient may have (e.g. vomiting, abdominal pain). For patients with intentional overdose, referral to psychiatry will be made upon admission to the EDOU. They will then be reviewed by psychiatry on the day of admission if admitted within office hours or the following morning if admitted after office hours. Patients are reviewed by psychiatry for continuing risk of self-harm and need for further psychiatric follow-up and care. For patients admitted to both ward and EDOU, those with high risk of further self-harm are then transferred to a psychiatric facility following completion of all medical treatment as decided by the psychiatric team.

Repeat liver function tests, coagulation studies and serum acetaminophen levels were taken at the end of treatment to determine adequacy of treatment and disposition. As the ED observation unit only allows admission for up to 48 h with protocolised care, patients who develop other medical issues requiring subspecialty management will be transferred to the ward under the relevant medical discipline for further management. Also, patients admitted to both the emergency observation unit and ward with significant risk of self-harm will be transferred to the national psychiatric hospital upon resolution of their medical issues. While no routine follow-up visit is arranged for the patients discharged from EDOU, all patients are given a standard verbal and written discharge advice advising them to return if they feel unwell following the discharge.

### Selection of patients into study

Electronic medical records of all patients who were admitted from the ED to either the general ward or the EDOU with a diagnosis of paracetamol/acetaminophen poisoning from 1 January 2012 till 31 December 2016 were reviewed. Patients who were admitted directly to the intensive care or high dependency unit, transferred to another hospital from the ED or discharged from the ED were excluded. Patients who fulfilled any exclusion criteria of the EDOU were also excluded from the final analysis. These included patients less than 16 years of age, pregnant patient, presence of other medical conditions that require inpatient management, agitated patients requiring sedation, unstable patients and patients already considered for transplant. Unstable patients were defined as having abnormal vital parameters (systolic blood pressure less than 90 mmHg, pulse oximetry less than 94% on room air) or depressed consciousness (Glasgow Coma score < =13).

### Data collection

Patient clinical data was abstracted from the hospital’s electronic record by a single investigator and recorded on a standard case report form. Cases are first reviewed for whether they meet the inclusion and/or exclusion criteria for EDOU admission under the acetaminophen treatment pathway (Table 2). Clinically significant acetaminophen overdose is defined as the ingestion of more than 150 mg/kg body weight acetaminophen tablets and/or require specific treatment for the overdose (i.e. *N*-acetylcysteine) based on clinician’s assessment.

**Table 2:** Characteristics of patients admitted who fulfil EDOU criteria for acetaminophen poisoning (*n* = 219)

Variables	Admitted EDOU (*n* = 153)	Admitted ward (*n* = 66)	*P*
Median age, years (IQR)	23 (19–32)	29 (19.8–35.5)	0.015
Gender			
Male, *n* (%)	38 (24.8)	27 (40.9)	0.017
Female, *n* (%)	115 (75.2)	39 (59.1)	
Ingestion			
Single, *n* (%)	121 (79.1)	51 (77.3)	0.76
Staggered, *n* (%)	32 (20.9)	15 (22.7)	
Median dose of paracetamol taken, g (IQR)	10 (9–15)	10.8 (8.6–15.8)	0.28
Given IV NAC, *n* (%)	124 (81.1)	55 (83.3)	0.69
Acetaminophen-induced liver injury			
No liver injury, *n* (%)	147 (96.0)	60 (90.9)	0.19
Developed acute liver injury^a^, *n* (%)	3 (2.0)	2 (3.0)	0.64
Developed hepatotoxicity^b^, *n* (%)	3 (2.0)	4 (6.1)	0.20

^a^AST/ALT double of initial value to at least 150 U/L

^b^AST/ALT increased to 1000 U/L and above

Other data abstracted include patient demographics, type of acetaminophen ingestion (single ingestion vs staggered ingestion), laboratory test results, whether *N*-acetylcysteine was given, final disposition from the hospital (discharge or transferred) and length of stay. Liver injury due to acetaminophen poisoning were defined into two categories—acute liver Injury is diagnosed if the aspartate aminotransferase (AST) or alanine aminotransferase (ALT) doubled from initial value to at least 150 U/L and hepatotoxicity is defined by the AST or ALT reaching 1000 U/L [14–16]. We also compared the length of stay in patients admitted to EDOU and ward who did not develop liver injury so as to adjust for the possible reason in the difference in length of stay that could be accounted by complications such as acute liver injury from acetaminophen poisoning.

For patients who were transferred from the EDOU to the inpatient medical unit, the case notes were reviewed for the reasons for the transfer. The electronic medical records of all patients included in the study were also reviewed for any return visits to the hospital within 7 days for any delayed complications arising from the overdose or treatment.

### Outcome measures

The primary outcome was the length of stay for the patients admitted to the EDOU and the general ward who fulfilled admission criteria for EDOU admission. Length of stay (LOS) was defined as the time from the admission of the patient to either the EDOU or the general ward until the discharge of the patient from the hospital. Thus, for patients admitted to EDOU and subsequently transferred to the ward, the length of stay includes their duration of stay in the wards till discharge from the hospital. Any differences in length of stay will therefore not be affected by bias arising from any administrative limitations on the length of stay in the EDOU.

Patients who discharged against medical advice or absconded are excluded from this analysis. Other outcomes were the incorrect utilisation of the observation unit admission and the failure of the emergency observation protocol.

Incorrect utilisation of the observation protocol comprise of two categories—over-utilisation and underutilisation. Over utilisation was defined as admission to the EDOU without either meeting the inclusion criteria or having one or more of the exclusion criteria. Underutilisation was defined as admission to the ward while meeting the admission criteria to the EDOU with no exclusion criteria. Failure of emergency observation protocol is defined as eventual admission of the patient to the general ward for further treatment after initial admission to the observation unit.

### Data analysis

The data was analysed using SPSS 19 (IBM Corp. Released 2010. IBM SPSS Statistics for Windows, Version 19.0. Armonk, NY: IBM Corp). Categorical data is presented in frequency with proportion to the total number of patients. For continuous data, test of normality was done using Kolmogorov-Smirnov test. Parametric data were presented as means with standard deviation while non-parametric data were presented as median with interquartile range. For comparison between categorical values in each independent sample, chi-square or fisher’s exact test was used as appropriate. For comparison between non-parametric continuous variables, Man-Whitney *U* test is used.

## Results

**Fig. 1:**
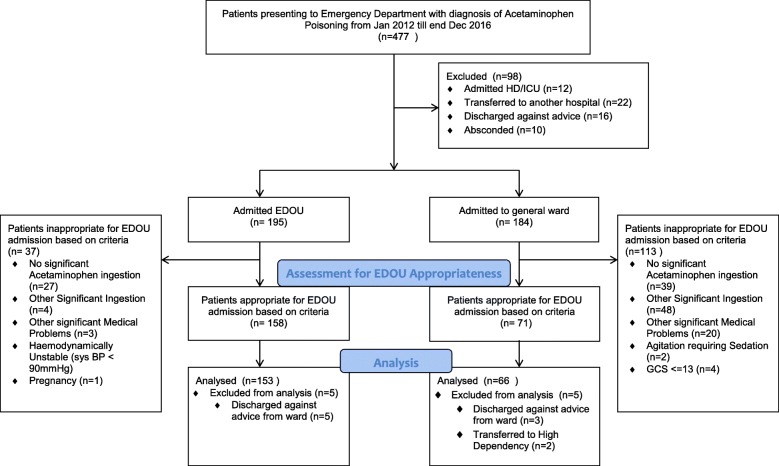
Flow diagram of patient selection into study

From 1 January 2012 till 31 December 2016, a total of 195 patients were admitted from the ED to the EDOU for acetaminophen poisoning and 184 patients were admitted to the general ward (Fig. 1).

Of the 195 patients admitted to the EDOU for acetaminophen poisoning, 158 patients (81%) meets the admission criteria. Twenty-seven (13.8%) patients did not have significant paracetamol ingestion despite admission under the pathway. Of the 184 patients admitted to the general ward, 71 patients (38.6%) meets admission criteria to EDOU. Among these 229 patients who fulfilled the criteria for EDOU admission, eight patients (five in observation unit and three in the general ward) discharged against medical advice before treatment were completed. A further two patients were admitted to the high dependency ward for complications related to acetaminophen poisoning and these ten patients were excluded from the final analysis.

**Fig. 2:**
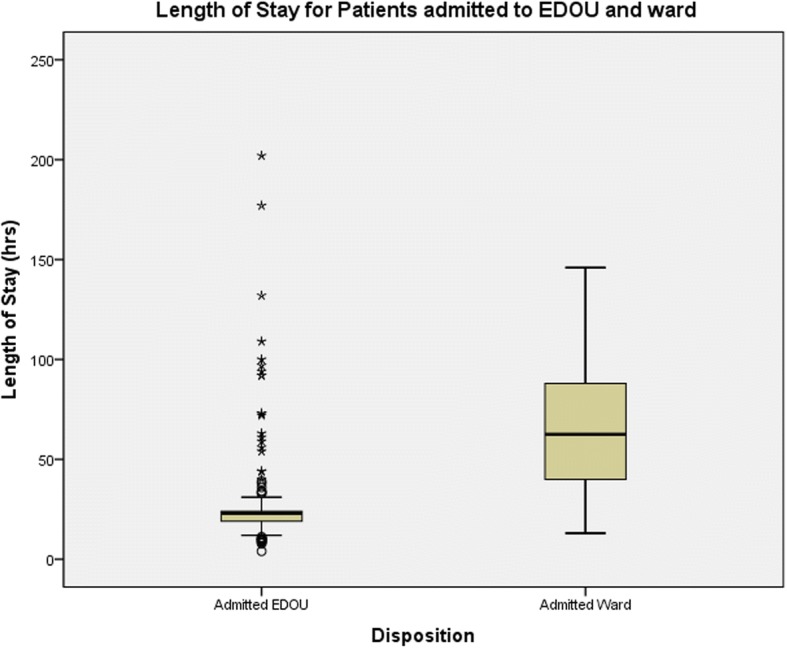
Length of stay for patients admitted for acetaminophen toxicity

The characteristics of patients who fulfilled the EDOU criteria and admitted to either EDOU or ward are shown in Table 2. Majority of the patients admitted for acetaminophen poisoning in both groups are young with a median age of 24 years and majority are female at 70.3%. 18.9% and 16.7% of patients admitted to the EDOU and ward, respectively, did not require *N*-acetylcysteine based on serum paracetamol levels but were admitted for observation of symptoms. There are no significant differences between the two groups with regards to the number of patients who were given IV NAC and who developed liver injury or toxicity. Outcomes of both groups of patients are shown in Table 3. Median LOS for EDOU patients was 23 h (IQR 19 to 24) while that for patients admitted to the ward was 66 h (IQR 62.5 to 88.3) (Fig. 2).

**Table 3:** Outcomes of patients who fulfil EDOU criteria for acetaminophen poisoning (*n* = 219)

Outcomes	Admitted EDOU (*n* = 153)	Admitted ward (*n* = 66)	*p*
Median, length of stay in hospital, h (IQR)	23 (19–24)	66 (62.5–88.3)	0.00
Transferred from observation ward to the general ward, *n* (%)	19 (12.4)		
Transferred to another hospital			
Psychiatric hospital, *n* (%)	8 (5.2)	7 (10.1)	0.39
Liver transplant unit, *n* (%)	1 (0.7)	0

**Fig. 3:**
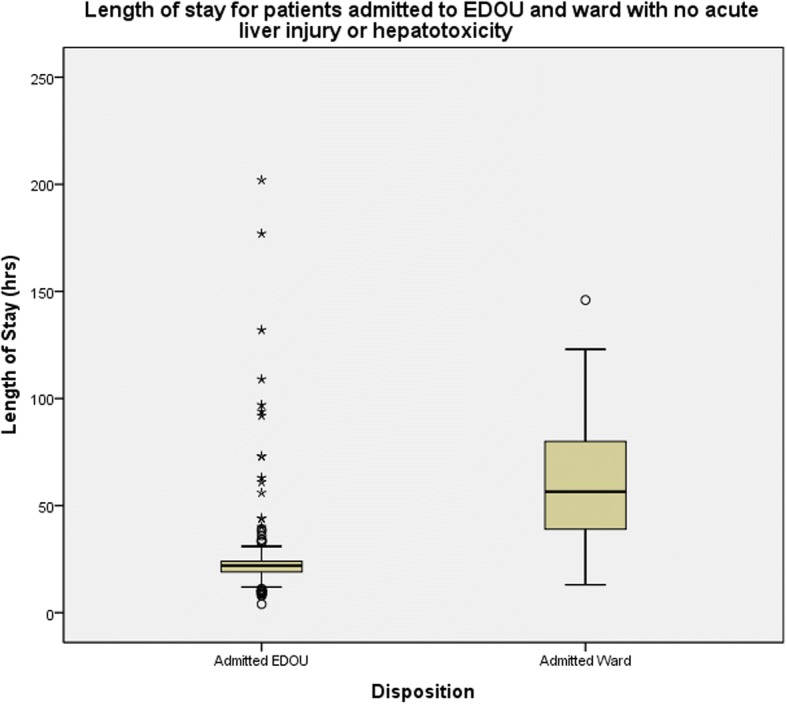
Length of stay for patients admitted for acetaminophen who did not develop liver injury

Duration of stay in EDOU is consistently shorter even when only patients without liver injury are considered. In this group of patients, median LOS for EDOU patients was 22 h (IQR 19 to 24) while that forward patients was 56.5 h (IQR 38.5 to 81) (Fig. 3).

Nine patients in the EDOU group (5.9%) and seven patients in the ward (10.1%) group were eventually transferred to another hospital. Most of the transfers were to a psychiatric facility due to high risk of suicide while one patient in the EDOU group was transferred to a hospital with transplant capability due to predicted poor prognosis. The patient eventually recovered without transplant. For the duration of hospital stay, 19 patients in the EDOU (12.4%) were transferred to the ward. The reasons for the transfer are shown in Table 4. Majority of the cases (52.6%) were transferred for further psychiatric monitoring and care. Among patients discharged from the EDOU there were no reattendance to the hospital within 7 days for any medical complications arising from the overdose or treatment while in hospital.

**Table 4:** Reasons for conversion from EDOU to ward (*n* = 19)

Reasons	Number of patients (%)
Abnormal laboratory tests (excluding liver Injury)	2 (10.5)
Acetaminophen induced liver injury	5 (26.3)
Psychiatric monitoring and care	10 (52.6)
Social issues	1 (5.3)

## Limitations

The main limitations of this study were related to this being a single-centre study and the inherent limitations of a retrospective design. There may also be bias due to the fact that one investigator extracted all the data; however, the variables used in this study are well defined and objective in nature. In the study clinical setting, the factors that the clinicians considered before determining if these patients should be admitted to EDOU or not were not documented in the case notes. These factors could be possible confounders that could have affected the length of stay. In this study, some clinical factors that may influence decision making and length of stay were eliminated by excluding patients who are unstable, mentally disturbed that require sedation or have other medical problems requiring intervention other than the overdose itself.

Another limitation of this study is that there were no follow-up arranged for the patients following discharge to ensure no adverse outcomes. However, all patients admitted to the EDOU were discharged with a standard discharge advice to return to the hospital if they feel unwell following discharge. The medical records were reviewed for any return visit to the hospital for complications related to the overdose or treatment. Despite this, any significant adverse events may be missed for patients who visited other hospitals or clinics subsequently.

In the study, patients admitted to the EDOU have early access to multi-disciplinary care including social work and psychiatric evaluation. All patients admitted to the ward and the EDOU that do not require further medical treatment but require psychiatric care are transferred to a separate psychiatric facility. That limits the applicability of this study to centres with such services.

## Discussion

This study demonstrates that the EDOU is an effective strategy in reducing the of length of stay for stable patients presenting with acute acetaminophen poisoning. This finding is consistent with other studies on management of poisoned patients in the EDOU [13, 17, 18]. Despite a proportion of patients being transferred to the ward from the EDOU due to persistent psychiatric or medical issues, the overall length of stay for patients admitted initially to EDOU were still significantly shorter than those admitted to the ward. There are some factors that contribute to this. Management of the patients follows a standardised care path and this facilitates the use of evidence-based medicine in optimising outcomes and improving clinical efficiency. Several studies have demonstrated that standardised care can reduce length of stay in certain conditions [19, 20]. In the EDOU, ward rounds are performed three times a day including weekends by a senior physician from the ED. With a large proportion of poisoned patients presenting out-of-office hours [21], more frequent review of ward patients at such hours prevents delays in management and deposition of these patients. As part of the standardised care in the EDOU, patients admitted for intentional acetaminophen poisoning are referred early to the psychiatry and social services as part of a multi-disciplinary approach. Such multi-disciplinary approach had been shown to reduce the length of stay for some acute medical conditions [22].

In our study, 12.4% of patients admitted to the EDOU were eventually transferred to the general ward which represents the failure of EDOU care. More than half of these patients are transferred for continual psychiatric care. This rate of inpatient transfer was comparable to previous studies on the outcomes of toxicology patients in EDOU [13, 18]. However, a proportion of patients (15.8%) were transferred because of medical and social issues not related to acetaminophen overdose. Hence, there is a need to further determine any predictive factors responsible for this failure of EDOU management to help better refine the admission criteria and the treatment protocol further. As the decision to admit these patients to EDOU was determined by the reviewing senior EP, there was a significant number of over-utilisation of the pathway whereby patients who did not meet the criteria for EDOU were admitted there and underutilisation whereby patients who met the criteria were admitted to the ward instead. While the reasons to determine if these patients should be admitted to EDOU or to inpatient medical team were not stated in the case notes, one possible reason could be the lack of familiarity with capabilities of the EDOU and understanding of the admission criteria. There may be a need to clarify some of the admission criteria to improve compliance rate.

From the study, medical treatment, i.e. IV NAC and development of hepatic injury, does not account for the differences in the length of stay between ward and EDOU as their frequency are similar in both groups of patients. In addition to the more frequent rounds and discharges in the EDOU, one possible factor in affecting the length of stay is the need for psychiatric care. The importance of psychiatric factors in affecting length of stay was also supported by the fact that majority of patients who failed EDOU treatment, as defined by transfer from EDOU to the inpatient ward, were for psychiatric care. Furthermore, most of the hospital transfers from the hospital were to a psychiatric facility due to persistent high risk of self-harm. Psychiatric disorders are prevalent in patients presenting with non-fatal self-harm, ranging from depression to substance misuse [23]. Zyoud et al. found that an acute depressed mood was an independent risk factor for a long length of stay in patients admitted for acetaminophen overdose [24]. Other studies have also shown an association between psychiatric comorbidity and increased length of stay for patients admitted for medical conditions [25, 26]. In the EDOU, the patients referred early upon admission to the psychiatric team and medical social workers as part of the multi-disciplinary approach and there was an arrangement with the psychiatric team for prioritisation of these patients for review to facilitate their disposition. Two studies had previously demonstrated that a proactive multi-disciplinary psychiatric consultation with close collaboration with the medical team for patients admitted for medical conditions is effective in decreasing the length of stay [27, 28]. A systemic review on consultation-liaison psychiatry services also suggests a shorter length of stay for medical patients with early referral to psychiatry [29].

## Conclusion

The EDOU is an effective alternative to ward hospitalisation in the management of acetaminophen poisoning. Our study suggests that the EDOU protocol is associated with a shorter length of stay for patients presenting with acetaminophen overdose. No patients re-attended the hospital for any complications following discharge from EDOU as well. Further studies are needed to investigate the cost-effectiveness of the EDOU protocol.

## Abbreviations

ALT: Alanine aminotransferase
AST: Aspartate aminotransferase
ED: Emergency department
EDOU: Emergency Department Observation Unit
EP: Emergency physician
IQR: Interquartile range
IV: Intravenous
LOS: Length of stay
NAC: *N*-Acetylcysteine
